# Seasonal and Temporal Patterns of Brace-Wearing Behavior in Adolescent Idiopathic Scoliosis Assessed Using Objective Sensor Data

**DOI:** 10.3390/medicina62081484

**Published:** 2026-08-01

**Authors:** Manuel Gahleitner, Michaela Winkler, Manuel Leitner, Regina Strassl, Matthias Holzbauer, Tobias Gotterbarm, Lorenz Pisecky

**Affiliations:** 1 Department for Orthopedics and Traumatology, Johannes Kepler University Linz, Krankenhausstraße 9, 4020 Linz, Austria; 2 Kepler University Hospital GmbH, Johannes Kepler University Linz, Altenberger Strasse 96, 4040 Linz, Austria; 3 Orthovida Plus GmbH, Rohrbacherstraße 2, 4111 Walding, Austria

**Keywords:** adolescent idiopathic scoliosis, brace therapy, temperature sensor, seasonal patterns, conservative management of scoliosis, brace adherence

## Abstract

*Background and Objectives*: Brace treatment is a key component of conservative management in adolescent idiopathic scoliosis (AIS), with treatment success strongly depending on patient adherence. While recommended wearing times are well defined, real-life adherence patterns remain insufficiently characterized. In particular, temporal and seasonal variations in brace-wearing behavior under real-world conditions have not yet been comprehensively characterized. *Materials and Methods*: This retrospective observational study analyzed objectively measured brace-usage data from scoliosis patients treated with temperature sensor-equipped braces. The study focused exclusively on objectively measured brace-wearing behavior; no clinical or radiographic outcome measures were included. Daily adherence time was recorded and aggregated across 6892 monitoring days from 43 patients. Brace wear was defined using a predefined temperature threshold (≥29 °C). Temporal patterns were assessed by analyzing weekday versus weekend differences, seasonal variation, and hourly wear probability. A linear mixed-effects model with patient as a random effect was applied to evaluate the influence of season, day type, and time since treatment initiation. *Results*: The mean objectively measured daily brace-wearing time was 9.22 h (median 8.67 h), demonstrating substantial variability. Wear time was slightly lower on weekends compared to weekdays (−0.34 h/day, *p* = 0.026). Seasonal differences in objectively measured brace-wearing time were observed (*p* < 0.001), with the highest values occurring during summer and the lowest during winter. Mixed-effects modeling confirmed increased wear time in summer (+2.05 h/day, *p* < 0.001) and reduced wear in winter (−1.27 h/day, *p* < 0.001) compared to spring. Heatmap analysis revealed pronounced diurnal patterns, with peak usage during nighttime hours (approximately 21:00–06:00) and reduced daytime adherence. A small but significant decline in wear time over the observation period was noted. *Conclusions*: Objective monitoring demonstrated temporal and seasonal variation in brace-wearing behavior under real-world conditions. Although environmental influences on temperature-based monitoring cannot be excluded, these findings provide valuable insights into adherence patterns and may support individualized patient counseling and future optimization of conservative scoliosis management.

## 1. Introduction

Adolescent idiopathic scoliosis (AIS) is a three-dimensional spinal deformity that affects approximately 2–3% of children and adolescents worldwide. For patients with progressive curves during growth, brace treatment remains the mainstay of non-operative management and has been shown to reduce the risk of curve progression and the need for surgical intervention when applied appropriately [1,2,3]. Despite well-established indications and treatment protocols, the effectiveness of brace therapy is highly dependent on patient adherence to the recommended wearing time [4]. Brace adherence has long been recognized as one of the most critical and challenging aspects of conservative scoliosis treatment. Traditionally, brace usage has been assessed through patient self-reports or parental estimates, which are known to be subject to recall bias and systematic overestimation [5,6]. Several studies have demonstrated substantial discrepancies between reported and actual brace-wearing time, highlighting the limitations of subjective assessment methods [7]. As a consequence, the interpretation of treatment outcomes and the identification of factors influencing brace effectiveness have been hampered by the lack of reliable adherence data.

The introduction of temperature- and pressure-based sensors embedded in scoliosis braces has enabled objective and continuous monitoring of usage in real-life conditions. These technologies have provided novel insights into actual wearing behavior and have revealed wide interindividual variability in adherence, even among patients treated within standardized protocols [8,9,10]. Objective monitoring has also been shown to improve brace wear in some settings, likely by increasing patient awareness and facilitating more targeted clinical counseling [11]. However, while the relationship between overall brace-wearing time and radiographic outcomes has been investigated in several landmark studies, including the BrAIST trial, less attention has been paid to temporal patterns of brace use in daily life [3,12]. Brace wear is not a static behavior but rather a dynamic process influenced by multiple factors, including physical comfort, psychosocial aspects, daily routines, and environmental conditions. Seasonal variation, ambient temperature, and daily or weekly schedules may all affect a patient’s willingness or ability to wear a brace for the prescribed duration. Previous qualitative studies and patient surveys have suggested that brace wear may decrease during warmer months or on weekends and holidays, when daily routines differ from school days [13,14]. However, these observations are largely based on subjective reports and have not been systematically evaluated using objective monitoring data.

Understanding temporal and seasonal patterns of brace-wearing behavior is clinically relevant for several reasons. First, identifying periods of reduced adherence may help clinicians tailor counseling strategies and follow-up schedules more effectively. Second, awareness of predictable adherence fluctuations may improve the interpretation of brace treatment outcomes, particularly in studies that rely on aggregated wearing time. Finally, knowledge of real-life patterns of usage can inform future brace design and patient education strategies aimed at improving comfort and long-term adherence.

The present study aims to analyze brace-wearing behavior in a cohort of scoliosis patients using objective, sensor-based daily wear data, with a specific focus on seasonal and temporal variables. By examining variations in brace wear across different seasons, weekdays versus weekends, and over time, this study seeks to provide an unbiased characterization of real-world adherence behavior. Given the absence of radiographic outcome data, the focus of this investigation is deliberately placed on adherence patterns rather than treatment effectiveness. We hypothesize that brace-wearing time differs significantly between seasons and across the weekly cycle, reflecting the influence of environmental and lifestyle-related factors on adherence. By providing a detailed analysis of objectively measured brace-wearing behavior, this study aims to contribute to a more nuanced understanding of adherence in scoliosis treatment and to support the development of patient-centered strategies to optimize conservative management.

## 2. Materials and Methods

This study was designed as a retrospective observational cohort study analyzing objectively measured brace-wearing behavior in patients undergoing brace treatment for scoliosis. The primary aim was to evaluate temporal and seasonal patterns of brace-wearing time based on daily sensor-derived wear data. No radiographic or clinical outcome measures were included, and the focus of the analysis was exclusively on objectively measured brace-wearing behavior. The study was conducted in accordance with the principles of the Declaration of Helsinki. All data were analyzed in anonymized form. Given the retrospective nature of the study and the exclusive use of routinely collected adherence data, formal informed consent was waived in accordance with local regulatory requirements.

**Figure 1:**
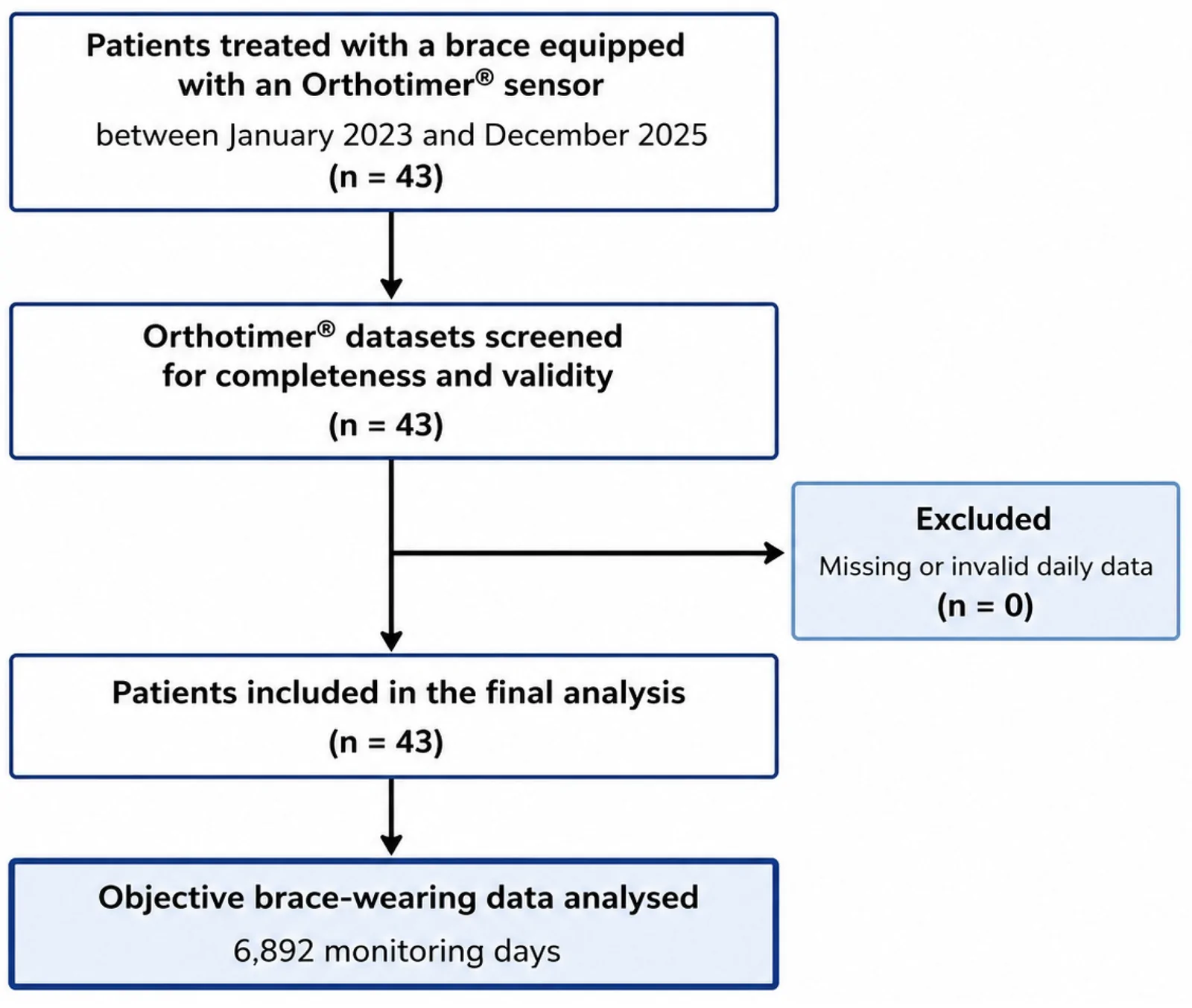
Flow diagram of patient selection for the final analysis.

All patients treated with a brace equipped with an embedded compliance-monitoring sensor, Orthotimer^®^ (Rollerwerk Medical Engineering & Consulting, Balingen, Germany), at a single center were screened for inclusion (Figure 1).

Patients were eligible for analysis if at least one full day of valid brace-wearing data was available. Each embedded sensor was uniquely assigned to a single brace and patient and served as a unique pseudonymized patient identifier. The study was based exclusively on an anonymized export of Orthotimer^®^ sensor data. The exported dataset contained objectively recorded brace-wearing times and corresponding timestamps only. No linkage to medical records or radiographic databases was performed. Consequently, demographic and clinical variables such as age, sex, BMI, Cobb angle, Risser stage, curve pattern, prescribed brace wear, treatment duration, and brace type were not available for analysis.

A temperature threshold of 29 °C was used to define active brace wear. This threshold is consistent with previously published validation studies demonstrating that temperature thresholds between 28 and 30 °C accurately distinguish brace wear from non-wear in scoliosis brace monitoring [15,16].

No additional clinical eligibility criteria were applied beyond the availability of at least one complete day of valid sensor data. As the analysis was based exclusively on anonymized Orthotimer^®^ sensor recordings, no selection was performed according to demographic or disease-specific characteristics.

The observational period varied between patients depending on treatment duration and data availability.

Brace-wearing time was recorded using an embedded sensor system integrated into the brace. The sensor continuously monitored brace usage and generated daily summaries of total wearing time, expressed in hours per day. Data were exported from the monitoring system and aggregated at the level of individual calendar days.

For each patient, daily brace-wearing time was linked to the corresponding calendar date. Seasons were categorized according to meteorological definitions as follows: winter (December–February), spring (March–May), summer (June–August), and autumn (September–November). In addition, each day was classified as either a weekday (Monday–Friday) or weekend day (Saturday–Sunday) to allow for analysis of weekly patterns.

All data were checked for plausibility and completeness prior to analysis. The exported dataset contained 6892 monitoring days from 43 patients. No missing dates, missing wear-time values, or implausible daily wear-time values outside the range of 0 to 24 h were identified. Accordingly, all 6892 monitoring days were included in the analysis, corresponding to 0% missing or excluded observations. No data imputation was performed.

The primary outcome measure was objectively measured daily brace-wearing time, expressed in hours per day. Secondary outcome measures included:

- Mean and median daily brace-wearing time per patient;
- Variability of brace wear across seasons;
- Differences in brace-wearing time between weekdays and weekends;
- Longitudinal trends in brace-wearing behavior over the observation period.

### Statistical Analysis

Statistical analyses were performed using IBM SPSS Statistics (version 26; IBM Corp., Armonk, NY, USA). All analyses were conducted on anonymized data. Descriptive statistics were used to summarize patient-level and day-level brace-wearing data. Continuous variables are reported as mean and standard deviation or median and interquartile range (IQR), as appropriate based on data distribution. Categorical variables are presented as counts and percentages.

For descriptive, unadjusted comparisons of brace-wearing time across seasons, Kruskal–Wallis tests were performed. The linear mixed-effects model served as the primary inferential analysis, accounting for the hierarchical structure of repeated daily measurements within patients. To account for the hierarchical structure of the data, with repeated daily measurements nested within individual patients, a linear mixed-effects model was fitted with daily brace-wearing time as the dependent variable. Season, day type (weekday versus weekend), and time since the start of monitoring were included as fixed effects, while patient was included as a random intercept to account for within-patient correlation.

The random-effects covariance structure consisted of a single variance component for the patient-specific random intercept. No additional covariance structure for repeated observations was specified.

Model parameters were estimated using restricted maximum likelihood (REML). Regression coefficients with 95% confidence intervals and two-sided *p* values are reported for all fixed effects. Random-intercept variance, residual variance, and the intraclass correlation coefficient (ICC) were calculated to quantify between-patient variability. Estimated marginal means were calculated for each season. Model assumptions were assessed by visual inspection of residual-versus-fitted and quantile–quantile plots.

To evaluate diurnal and weekly patterns of brace use, a heatmap analysis was performed based on hourly wear probability. For each calendar day, brace wear was dichotomized at the hourly level (worn vs. not worn) according to the predefined temperature threshold. The proportion of wear per hour was then calculated across all patients and aggregated by weekday (Monday–Sunday) and hour of the day (0–23). Mean hourly wear probability was visualized as a two-dimensional heatmap, with color intensity representing the percentage of patients wearing the brace at a given weekday-hour combination. This descriptive analysis was used to identify temporal clustering of brace use across the weekly cycle.

All statistical tests were two-sided, and a *p* value < 0.05 was considered statistically significant. Statistical analyses were performed using standard statistical software.

## 3. Results

A total of 43 patients were included in the analysis. Objective brace-wearing data were available for 6892 individual days of monitoring between the beginning of 2023 and the end of 2025. Monitoring duration varied between patients, with a median observation period of 148 days (IQR 68–235 days; range 1–405 days), reflecting real-world differences in treatment duration and data availability. Across the entire cohort, the mean objectively measured daily brace-wearing time was 9.22 h (±7.52), with a median of 8.67 h per day.

**Figure 2:**
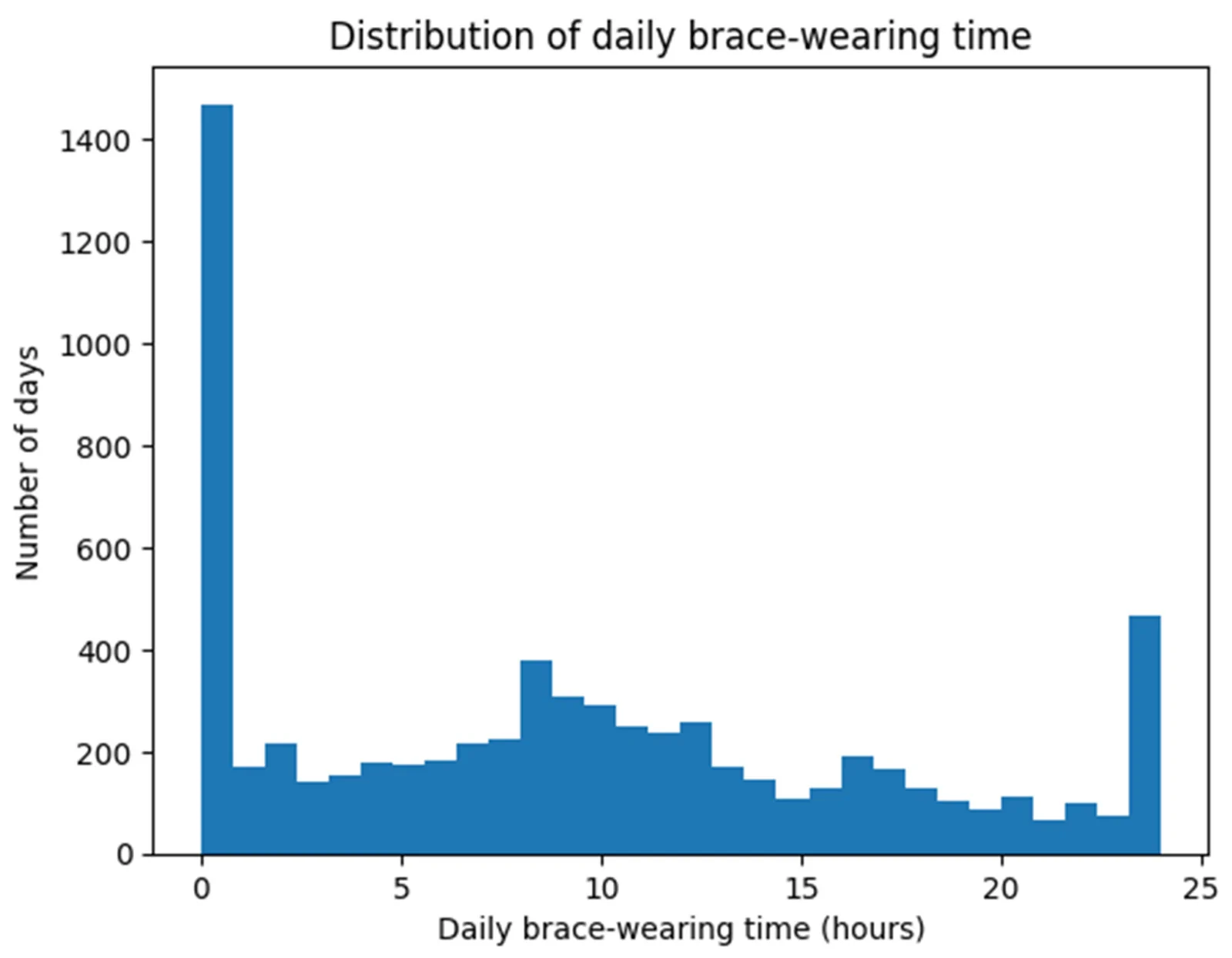
Histogram of objectively measured daily brace-wearing time across all monitoring days.

**Figure 3:**
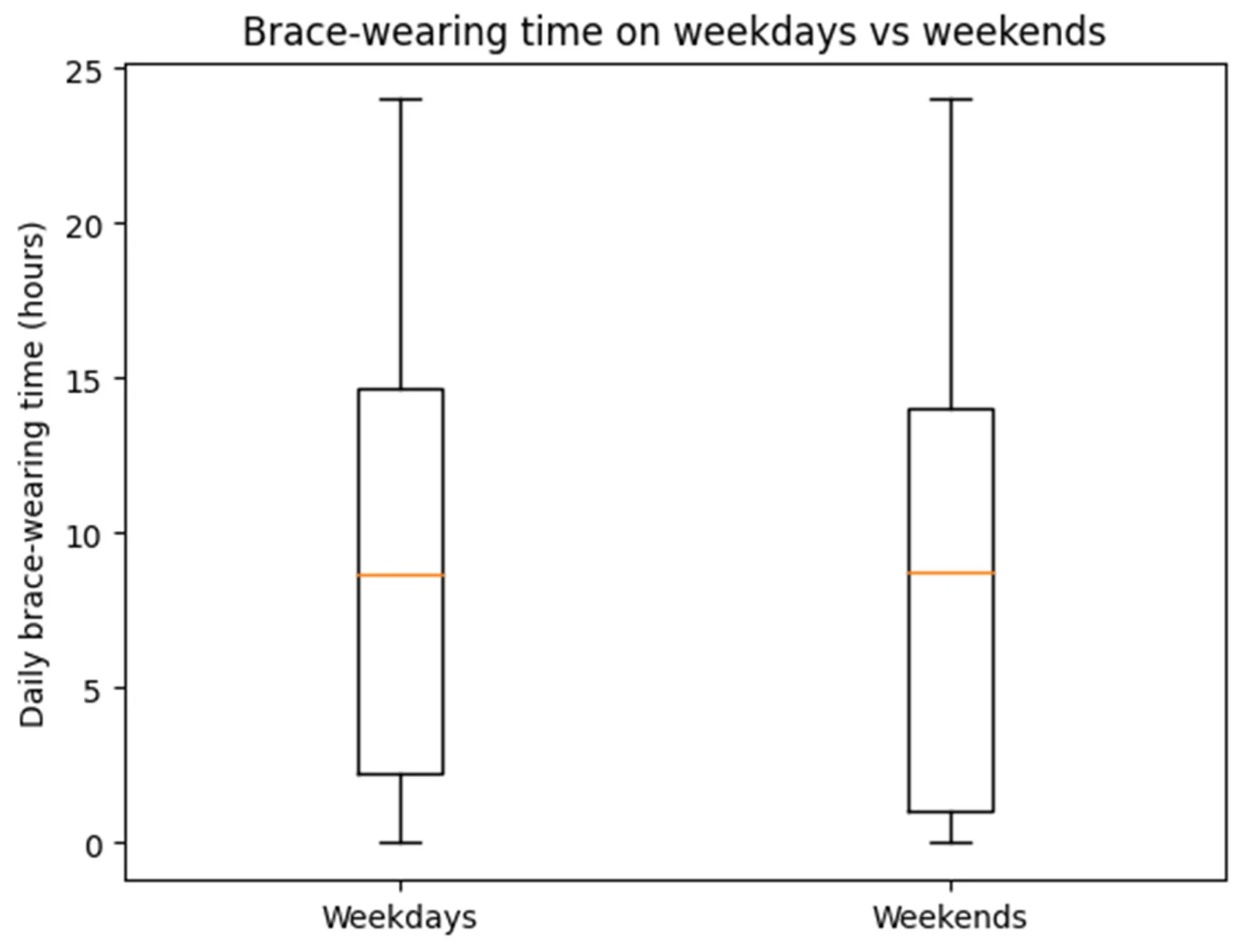
Boxplots of daily brace-wearing time on weekdays and weekends.

**Figure 4:**
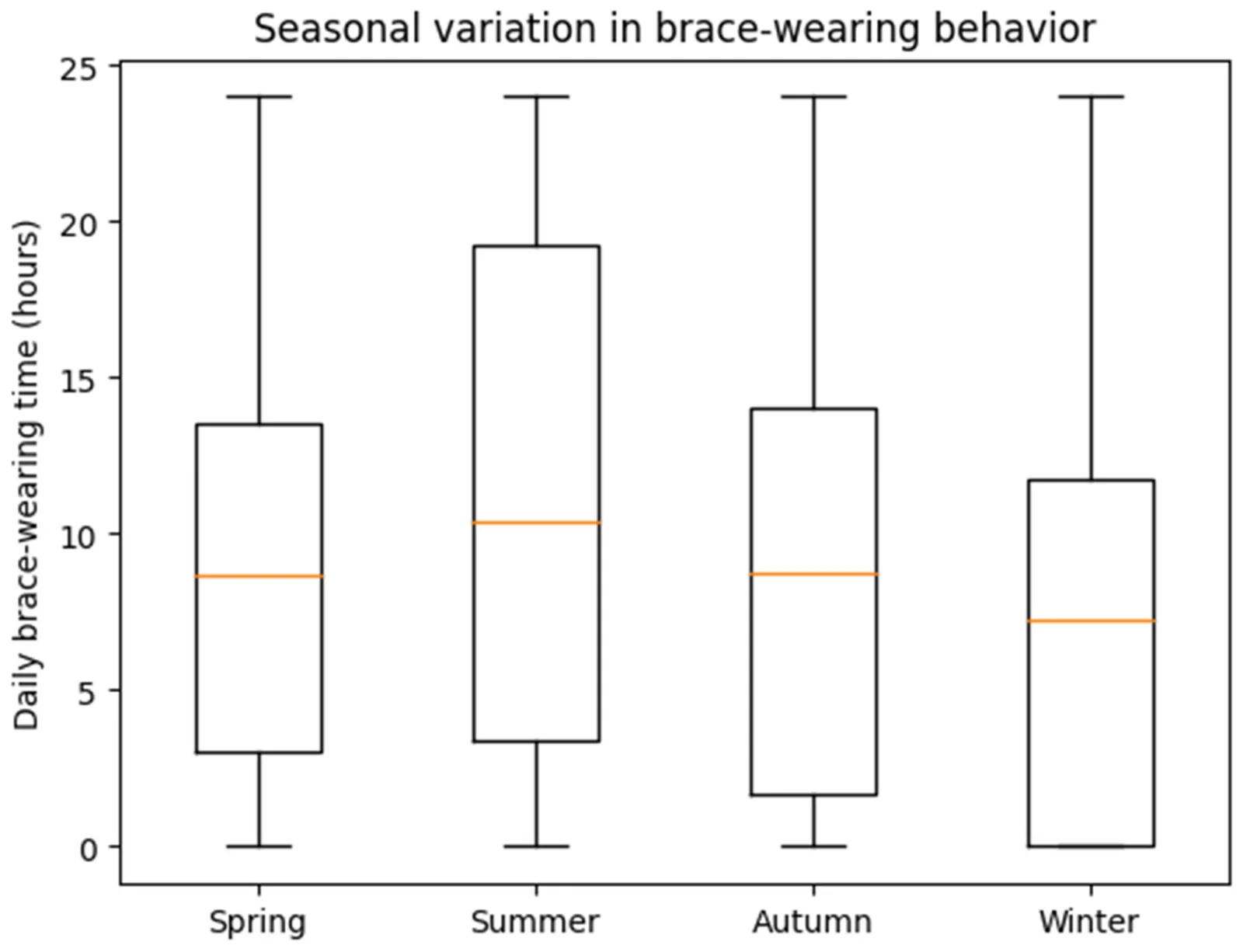
Boxplots of daily brace-wearing time stratified by season.

Brace wear demonstrated substantial variability, as reflected by an interquartile range (IQR) of 12.5 h, indicating marked inter- and intra-individual differences in adherence behavior. Figure 2 shows the distribution of objectively measured daily brace-wearing time across all monitored days. Brace-wearing behavior differed between weekdays and weekends. On weekdays (Monday to Friday), patients wore their braces for a mean of 9.31 h per day, whereas on weekend days (Saturday and Sunday), mean daily wear time decreased to 8.99 h (Figure 3). Median brace-wearing time was comparable between weekdays (8.67 h/day) and weekends (8.75 h/day), although overall wear time tended to be slightly reduced during weekends. Marked differences in brace-wearing time were observed across seasons. The highest mean daily brace usage was recorded during the summer months, with a mean of 11.30 h per day and a median of 10.42 h per day. In contrast, the lowest brace-wearing time was observed during winter, with a mean of 7.40 h per day and a median of 7.25 h per day (Figure 4). Brace-wearing time differed significantly across seasons (Kruskal–Wallis test, *p* < 0.001). In contrast, although brace wear tended to be lower during weekends compared to weekdays, this difference did not reach statistical significance (*p* = 0.065). Intermediate values were observed in spring (mean: 9.16 h/day, median: 8.67 h/day) and autumn (mean: 8.78 h/day, median: 8.75 h/day). Seasonal differences were consistent across the cohort and were observed over a large number of monitoring days in each season.

**Figure 5:**
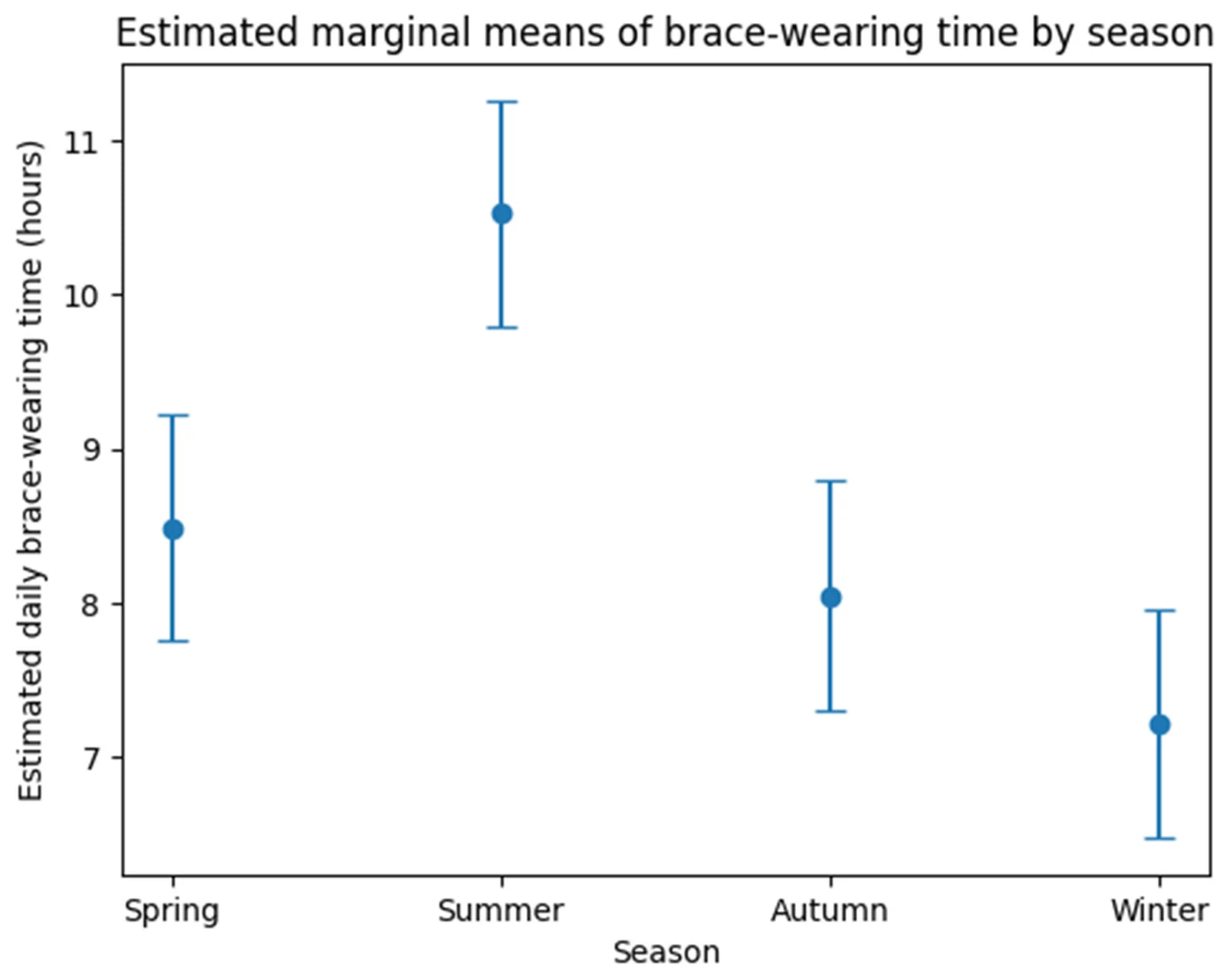
Adjusted mean daily brace-wearing time by season derived from the linear mixed-effects model. Error bars indicate standard errors.

In the linear mixed-effects model, patient was included as a random intercept to account for repeated daily measurements within individuals. The random-intercept variance was 21.31 and the residual variance was 32.40, yielding an intraclass correlation coefficient (ICC) of 0.397. Brace-wearing time differed significantly across seasons. Using spring as the reference category, daily brace wear was 2.05 h higher during summer (95% CI, 1.65 to 2.45; *p* < 0.001) and 1.27 h lower during winter (95% CI, −1.67 to −0.87; *p* < 0.001). No significant difference was observed between autumn and spring (β = −0.43 h/day; 95% CI, −0.92 to 0.05; *p* = 0.081). Compared with weekdays, brace wear was modestly reduced on weekends by 0.34 h per day (95% CI, −0.64 to −0.04; *p* = 0.026). A small but statistically significant decline in brace-wearing time over the monitoring period was also observed (β = −0.0026 h/day; 95% CI, −0.0040 to −0.0013; *p* < 0.001). Post hoc pairwise comparisons with Holm adjustment confirmed significantly higher brace-wearing time during summer compared with spring, autumn, and winter (all adjusted *p* < 0.001) (Figure 5, Table 1).

**Figure 6:**
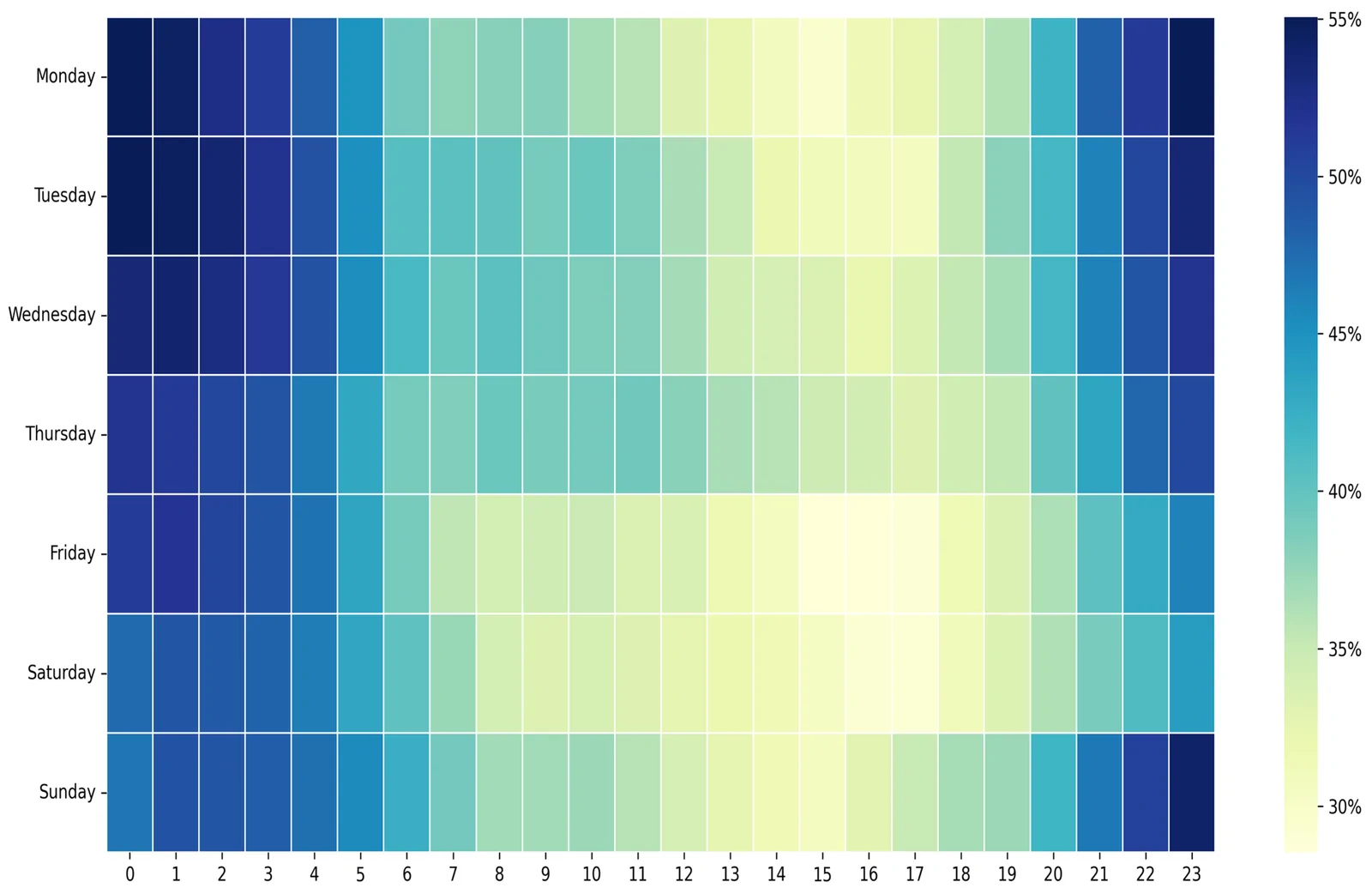
Heatmap of hourly brace-wearing probability by weekday. Color intensity represents the proportion of recorded hours classified as brace wear (>29 °C).

Estimated marginal mean brace-wearing time was 10.53 h/day (95% CI 9.10–11.97) during summer, 8.49 h/day (95% CI 7.05–9.92) during spring, 8.05 h/day (95% CI 6.59–9.52) during autumn, and 7.22 h/day (95% CI 5.77–8.66) during winter. The heatmap analysis revealed a pronounced diurnal pattern of brace wear across the weekly cycle. Wear probability was highest during late evening and nighttime hours (approx. 9:00 p.m.–06:00 a.m.) reaching peak values of approximately 50–55%. In contrast, the lowest wear probability was observed during midday and early afternoon hours (approx. 12:00–4:00 p.m.), with values around 30%. Weekday patterns were largely consistent from Monday to Thursday, with slightly reduced wear probability observed on Fridays and Saturday during daytime hours. On Sundays, evening wear appeared particularly pronounced, approaching the highest observed probabilities. Overall, brace use demonstrated a clear clustering during nocturnal hours with reduced daytime adherence, independent of weekday (Figure 6).

## 4. Discussion

The present study provides a detailed analysis of objectively measured brace-wearing behavior in a cohort of scoliosis patients, with a specific focus on temporal and seasonal patterns. Using sensor-based daily wear data, we observed substantial variability in brace adherence, clear differences between weekdays and weekends, and pronounced seasonal variation in brace-wearing time. These findings highlight the dynamic nature of brace adherence in real-life conditions and underscore the importance of considering temporal factors when evaluating conservative scoliosis treatment. Across the entire cohort, mean daily brace wear was approximately nine hours per day, which is notably lower than commonly prescribed wearing times reported in clinical guidelines and previous outcome-driven studies [1,2,17]. However, this finding should be interpreted with caution. As the present study was based exclusively on anonymized Orthotimer^®^ sensor data, information on the prescribed daily brace-wearing time was not available. Consequently, it was not possible to distinguish between full-time and part-time bracing or to determine individual adherence relative to the prescribed treatment regimen. Therefore, the observed mean wearing time should not be interpreted as poor compliance but rather as the objectively measured average brace-wearing behavior within the study cohort.

This discrepancy between recommended and actual brace wear has been consistently reported in studies using objective monitoring and reflects the challenges of achieving high adherence in daily life [4,5,6]. The wide interquartile range observed in our cohort further emphasizes the heterogeneity of brace-wearing behavior, even within a single treatment setting.

An important consideration when interpreting this variability is that the present study was based exclusively on anonymized Orthotimer^®^ sensor data and was not linked to clinical or radiographic records. Consequently, demographic and disease-specific characteristics such as age, sex, body mass index, Cobb angle, Risser stage, curve pattern, prescribed brace wear, and treatment duration were not available for analysis. These factors may contribute to interindividual differences in brace-wearing behavior and therefore represent potential sources of heterogeneity. Among these factors, patient age may be of particular relevance. Younger adolescents may benefit from greater parental supervision and structured daily routines, whereas older adolescents often experience increasing autonomy, social activities, and treatment fatigue, all of which may influence brace-wearing behavior. Future prospective studies combining objective adherence monitoring with clinical data are needed to investigate these potential age-related differences.

However, the primary objective of this study was to characterize temporal patterns of objectively measured brace wear rather than to identify patient-related predictors of adherence or treatment outcome.

Brace-wearing time was slightly reduced during weekends compared to weekdays. Although the absolute difference was modest, this pattern was consistent across the cohort and aligns with previous observations suggesting that structured daily routines, such as school attendance, facilitate more consistent brace use [18]. Weekends are typically associated with greater variability in daily activities, social engagements, and leisure pursuits, which may interfere with brace wear. Objective measured brace wear is also likely influenced by psychosocial and practical factors that were beyond the scope of the present study. Body image concerns, peer acceptance, teasing or bullying, participation in sports, and challenges related to prolonged brace wear, such as dressing or personal hygiene, may all affect the willingness to wear a brace consistently. Although these factors were not available for analysis, they represent important determinants of adherence and should be considered during patient counselling and in future prospective investigations.

From a clinical perspective, this finding suggests that counseling strategies should explicitly address weekend adherence and encourage patients to maintain consistent wearing habits beyond school days. Although the difference reached statistical significance, the absolute reduction in brace wear was modest (approximately 20 min per day). Therefore, its direct clinical relevance is likely limited when considered in isolation. Rather than indicating a clinically meaningful reduction in treatment adherence, this finding may reflect a consistent behavioral pattern associated with changes in daily routine between weekdays and weekends. As the present study did not evaluate radiographic outcomes, no conclusions can be drawn regarding the potential impact of this difference on treatment effectiveness.

One of the most interesting findings of this study was the observed seasonal variation in brace-wearing behavior. In the present cohort, objectively measured brace-wearing time was highest during summer and lowest during winter. Although this finding differs from the commonly held assumption that brace wear decreases during warmer months, it should be interpreted cautiously, as both behavioral factors and a potential influence of ambient temperature on temperature-based sensor measurements may have contributed to the observed seasonal differences.

Several factors may contribute to this unexpected finding. The observed seasonal differences are likely multifactorial and should not be attributed exclusively to environmental or weather-related influences. Seasonal changes in brace-wearing behavior may also reflect variations in school schedules, vacation periods, examination phases, sports participation, treatment stage, brace adjustments, and other lifestyle-related factors. As these variables were not available in the present dataset, their individual contribution could not be evaluated.

During summer months, school obligations are reduced, potentially allowing patients greater flexibility to compensate for brace-free periods by increasing wear during evenings or nights. Additionally, increased parental supervision during holidays may positively influence adherence. In contrast, winter months are often associated with increased clothing layers, reduced physical activity, and longer periods spent indoors, which might paradoxically reduce brace wear due to discomfort, restricted mobility, or decreased perceived necessity.

An important methodological aspect that warrants careful discussion is the use of temperature-based sensors to determine brace wear. The sensor employed in this study detects brace usage based on temperature thresholds approximating body temperature. While this technology has been widely validated and used in previous studies, it is inherently sensitive to environmental temperature conditions [8,10,11]. During warmer ambient conditions, particularly in summer, the temperature within the brace may approach or exceed the sensor’s activation threshold even when the brace is not worn continuously. This raises the possibility of an overestimation of brace wear during summer months. Conversely, during colder seasons, the sensor may underestimate wear time if thermal equilibration between the brace and the body is delayed or incomplete.

However, several factors mitigate the likelihood that the observed seasonal differences are solely attributable to sensor-related bias. First, the magnitude of the seasonal differences observed in this study exceeds what would be expected from minor temperature-related measurement fluctuations alone. Second, the consistency of the seasonal pattern across a large number of monitored days supports the robustness of the findings. Third, previous validation studies of temperature-based compliance monitors have demonstrated high accuracy across a wide range of environmental conditions when appropriate thresholds are applied [8,11].

Nevertheless, the potential influence of ambient temperature on sensor accuracy represents an important limitation of this study. Future investigations may benefit from combining temperature-based sensors with additional modalities, such as pressure or motion sensors, to further improve measurement precision. Alternatively, incorporating local ambient temperature data could allow for sensitivity analyses to better quantify the potential impact of environmental conditions on wear-time estimation. Although the present study included a large number of objectively recorded monitoring days, the cohort comprised only 43 individual patients. Therefore, the observed temporal patterns should be interpreted with appropriate caution, as the generalizability of the findings to the broader population of brace-treated patients with scoliosis remains limited. Confirmation in larger, preferably multicenter, cohorts is warranted.

Despite these limitations, the findings of this study have important clinical implications. The present findings suggest that brace-wearing behavior may vary across seasons and weekly routines. However, given the observational design and the potential influence of environmental temperature on sensor measurements, these findings should be interpreted with appropriate caution and confirmed in future prospective studies.

For example, increased attention to adherence during winter months or weekends may help mitigate predictable reductions in brace wear. Moreover, objective adherence data can facilitate more individualized patient discussions, moving beyond generalized assumptions toward data-driven counseling. Importantly, this study deliberately refrains from drawing conclusions regarding treatment effectiveness or radiographic outcomes. However, adherence behavior represents a prerequisite for any meaningful interpretation of treatment success. By providing an unbiased characterization of real-life brace wear, this study contributes essential contextual information for future outcome-focused research.

### Limitations

Several limitations must be acknowledged. First, this study was based exclusively on anonymized Orthotimer^®^ sensor data without linkage to clinical or radiographic records. Consequently, demographic and disease-specific variables, including age, sex, body mass index, Cobb angle, Risser stage, curve pattern, prescribed brace wear, treatment duration, and brace type, were unavailable. These factors may influence brace-wearing behavior and therefore could not be considered as potential confounders or covariates in the statistical analyses. The retrospective design and the absence of radiographic or clinical outcome data further preclude conclusions regarding the relationship between brace wear and curve progression. Additionally, the reliance on temperature-based sensors introduces the possibility of measurement bias related to environmental conditions, as discussed above. Finally, psychosocial factors and patient-reported comfort were also unavailable and may have influenced adherence behavior. Future prospective studies integrating objective compliance monitoring with comprehensive clinical and radiographic data are needed to better identify determinants of brace adherence. Furthermore, although the dataset comprised 6892 monitoring days, the effective sample size was limited to 43 patients. Consequently, the generalizability of the findings is limited, and the observed temporal patterns should be confirmed in larger, multicenter cohorts. Furthermore, information regarding the prescribed daily brace-wearing duration was unavailable. Therefore, individual adherence could not be evaluated relative to the intended treatment regimen. In addition, the inclusion criteria required only one valid day of brace-monitoring data. Although this approach maximized the use of available real-world data, it may have introduced heterogeneity because monitoring duration varied substantially between patients. Future studies should consider predefined minimum monitoring periods or sensitivity analyses restricted to patients with longer observation times.

## 5. Conclusions

This study demonstrates that brace-wearing behavior in scoliosis patients is highly variable, with evidence of temporal and seasonal variation. Objective sensor-based monitoring revealed reduced brace wear during weekends and pronounced seasonal differences, with higher wear during summer and lower wear during winter. While potential sensor-related measurement bias must be considered, these findings provide valuable insights into real-life adherence patterns and highlight the importance of incorporating temporal context into the management and evaluation of conservative scoliosis treatment.

## Figures and Tables

**Table 1:** Regression coefficients from the linear mixed-effects model. Spring and weekdays served as the reference categories. Random-intercept variance = 21.31; residual variance = 32.40; and ICC = 0.397. REML estimation was used.

Fixed Effect	β (h/day)	SE	95% CI	*p*-Value
Intercept (Spring, Weekday)	8.99	0.74	7.55–10.43	<0.001
Summer vs. Spring	+2.05	0.21	1.65–2.45	<0.001
Autumn vs. Spring	−0.43	0.25	−0.92–0.05	0.081
Winter vs. Spring	−1.27	0.20	−1.67–−0.87	<0.001
Weekend vs. Weekday	−0.34	0.15	−0.64–−0.04	0.026

## Data Availability

The data presented in this study are available upon request from the corresponding author. The collected data are properly stored in the study archive of the Department of Orthopaedics and Traumatology at Kepler University Hospital and protected from misuse by third parties.

## Abbreviations

The following abbreviations are used in this manuscript:

AIS	Adolescent idiopathic scoliosis
BrAIST	Bracing in Adolescent Idiopathic Scoliosis Trial
CI	Confidence interval
IQR	interquartile range
SD	Standard deviation
ICC	Intraclass correlation coefficient
